# Supplementary material for: Targeted Disruption of Bone Marrow Stromal Cell-Derived Gremlin1 Limits Multiple Myeloma Disease Progression In Vivo
Source: Cancers (Basel). 2020 Aug 3;12(8):2149. doi: 10.3390/cancers12082149 (PMC7464474; doi:10.3390/cancers12082149)
Supplement: Supplementary file 1 [file cancers-12-02149-s001.pdf]

Supplementary Materials: Targeted Disruption of Bone Marrow Stromal Cell-Derived Gremlin1 Limits Multiple Myeloma Disease Progression in Vivo

Kimberley C. Clark, Duncan R. Hewett, Vasilios Panagopoulos, Natalya Plakhova, Khatora S. Opperman, Alanah L. Bradey, Krzysztof M. Mrozik, Kate Vandyke, Siddhartha Mukherjee, Gareth C.G. Davies, Daniel L. Worthley, Andrew C.W. Zannettino

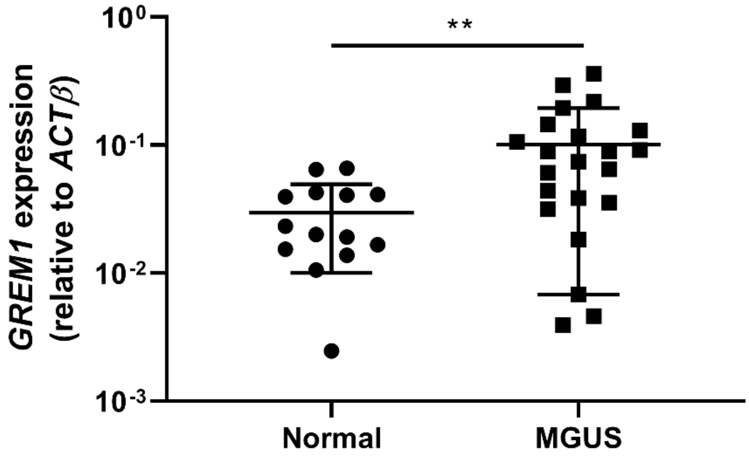

Figure S1. *GREM1* expression is elevated in primary stromal cultures from MGUS patients**.** RNA was extracted from stromal cells ex vivo cultured from BM trephine samples from age and gender matched normal donors and MGUS patients and the expression of *GREM1* was analysed by real-time PCR. Data presented as mRNA expression normalised to β-Actin (*ACTB*), mean ± SD, Normal; $n = 14$ and MGUS; $n = 22$, $^{**}p < 0.01$, Mann-Whitney test.

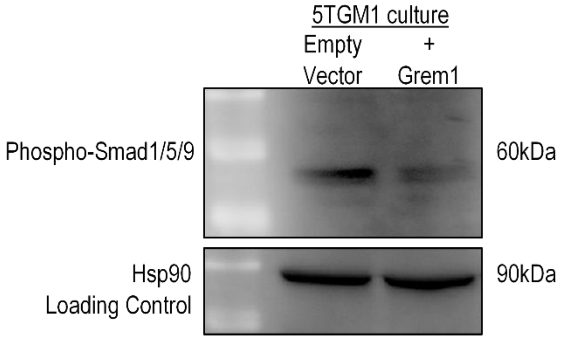

Figure S2: BMP signalling is downregulated in 5TGM1 MM PCs co-cultured with stromal cells overexpressing Grem1**.** 5TGM1 MM PCs were co-cultured in the presence of either a murine OP9 BM-stromal cell line overexpressing *Grem1*(+Grem1) or a vector-only control OP9 cell line (Empty Vector). Protein lysates from 5TGM1 MM PCs in co-culture were isolated and BMP signalling was analysed by Western blot for the downstream signalling of Phospho-Smad 1/5/9 compared to Hsp90 loading control.

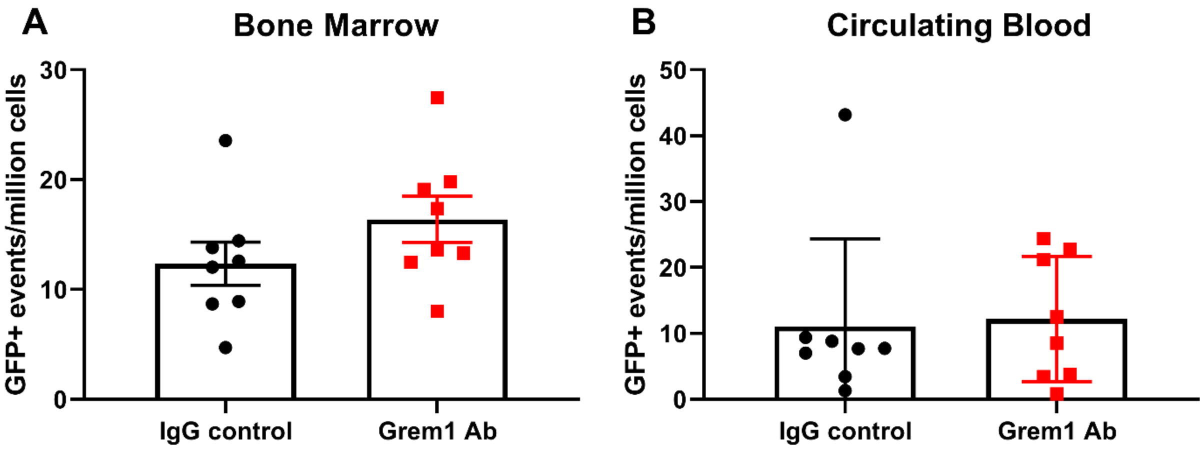

Figure S3. Blocking Grem1 activity with an anti-Grem1 neutralising antibody significantly reduces MM PC proliferation in co-culture with BM stroma but not in monocultures of MM PCs in vitro. 5TGM1.BMx1 MM PCs were cultured in the presence of 1µg/mL anti-Grem1 antibody or IgG isotype control antibody in the (A) absence or (B) presence of OP9 BM stromal cells and allowed to grow for 72hours. 5TGM1 proliferation was assessed by luciferase activity and results were normalised to the IgG control for replicate experiments. Mean ± SEM of $n = 3$ replicate experiments. ** $p < 0.01$, paired *t*-test.

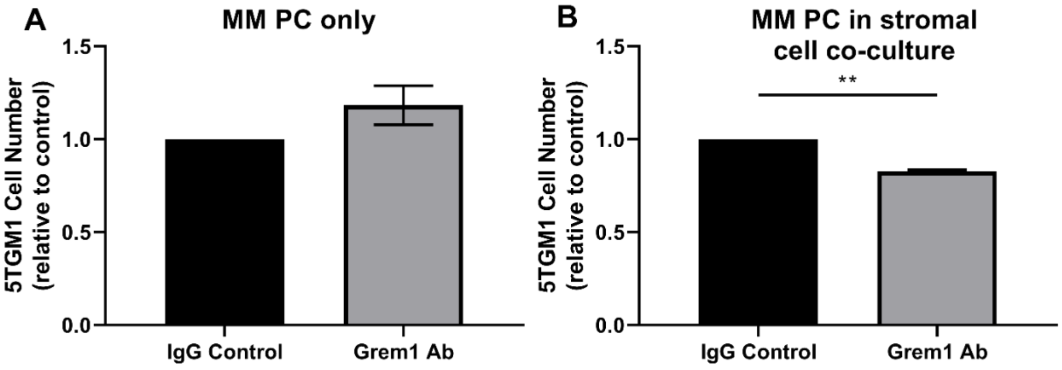

Figure S4. Anti-Grem1 antibody treatment does not alter the ability of 5TGM1 MM PCs to home to the BM in vivo**.** KaLwRij mice were treated with 30mg/kg anti-Grem1 antibody or IgG control twice over one week and subsequently injected with 5 x 10^6 5TGM1 MM PCs. GFP+ 5TGM1 MM PC within the **(A)** BM or **(B)** in the circulation 24 hours after tumour cell inoculation were analyzed by flow cytometry. Graphs depict mean ± SD of $n = 8$ per treatment group from two independent replicate experiments, $p > 0.05$, Mann-Whitney test.

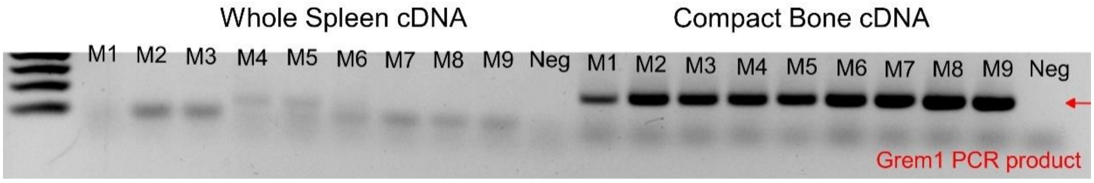

Figure S5: Differential *Grem1* expression in whole splenic and compact bone isolates**.** RNA from whole cell suspensions of the spleen and compact bone of KaLwRij mice ($n = 9$) was analysed by RT-PCR for *Grem1* mRNA expression. PCR products were visualized on a 2% agarose gel.

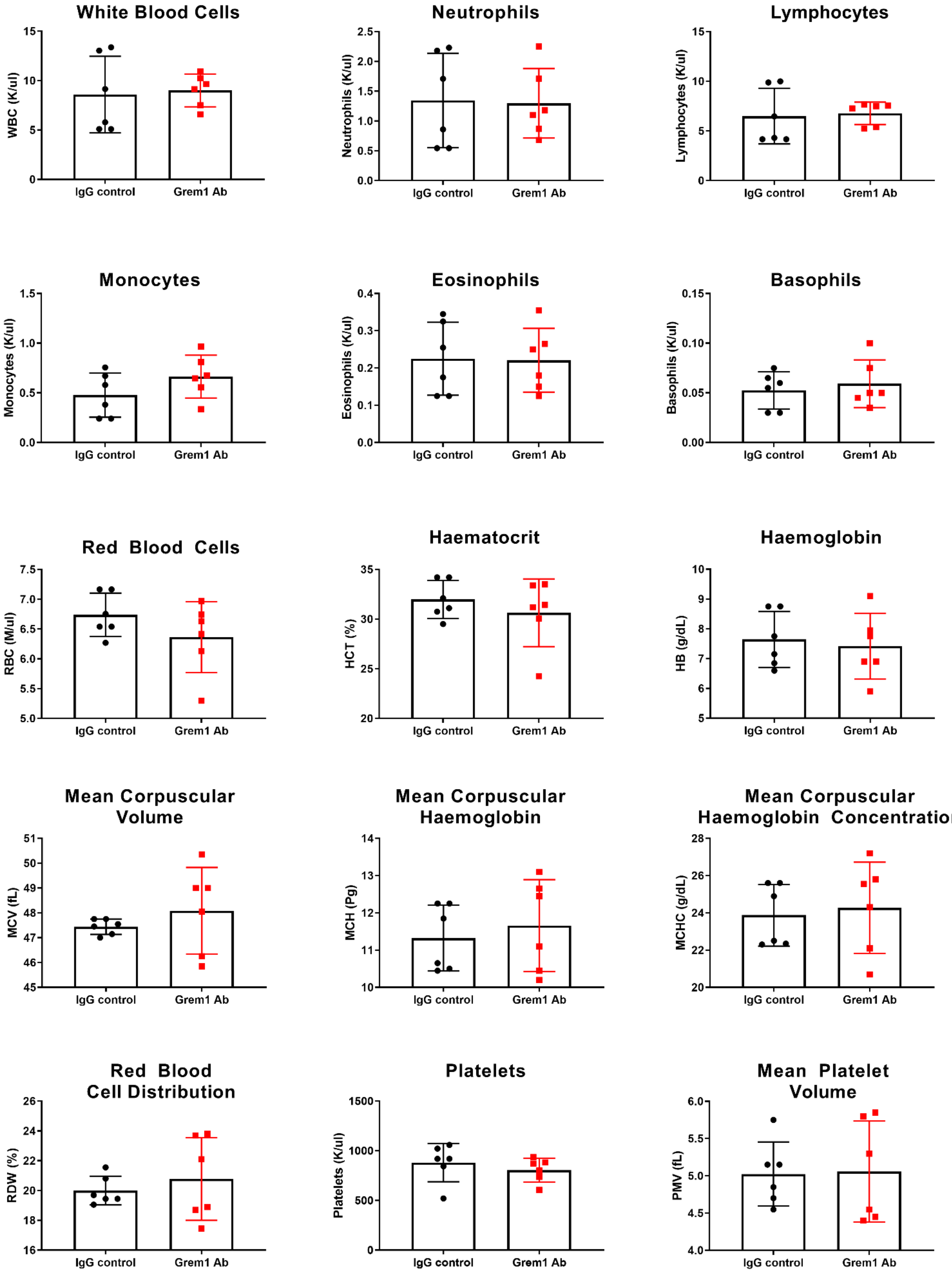

Figure S6. Anti-Grem1 treatment does not alter any haematological parameters in vivo. KaLwRij mice were treated with 30mg/kg anti-Grem1 antibody or IgG control twice weekly for two weeks. Peripheral blood was collected by cardiac puncture three days after the final treatment and was

analysed on a HEMAVET™ analyser. Graphs depict mean ± SD from $n = 6$ mice per treatment group from two independent experiments. $p > 0.05$, unpaired t-test. WBC=white blood cell, RBC=red blood cell, HB=haemoglobin, HCT=haematocrit, MCV=mean corpuscular volume, MCH=mean corpuscular haemoglobin, MCHC=mean corpuscular haemoglobin concentration, RDW=red blood cell distribution width, PLT=platelet, MPV=mean platelet volume.

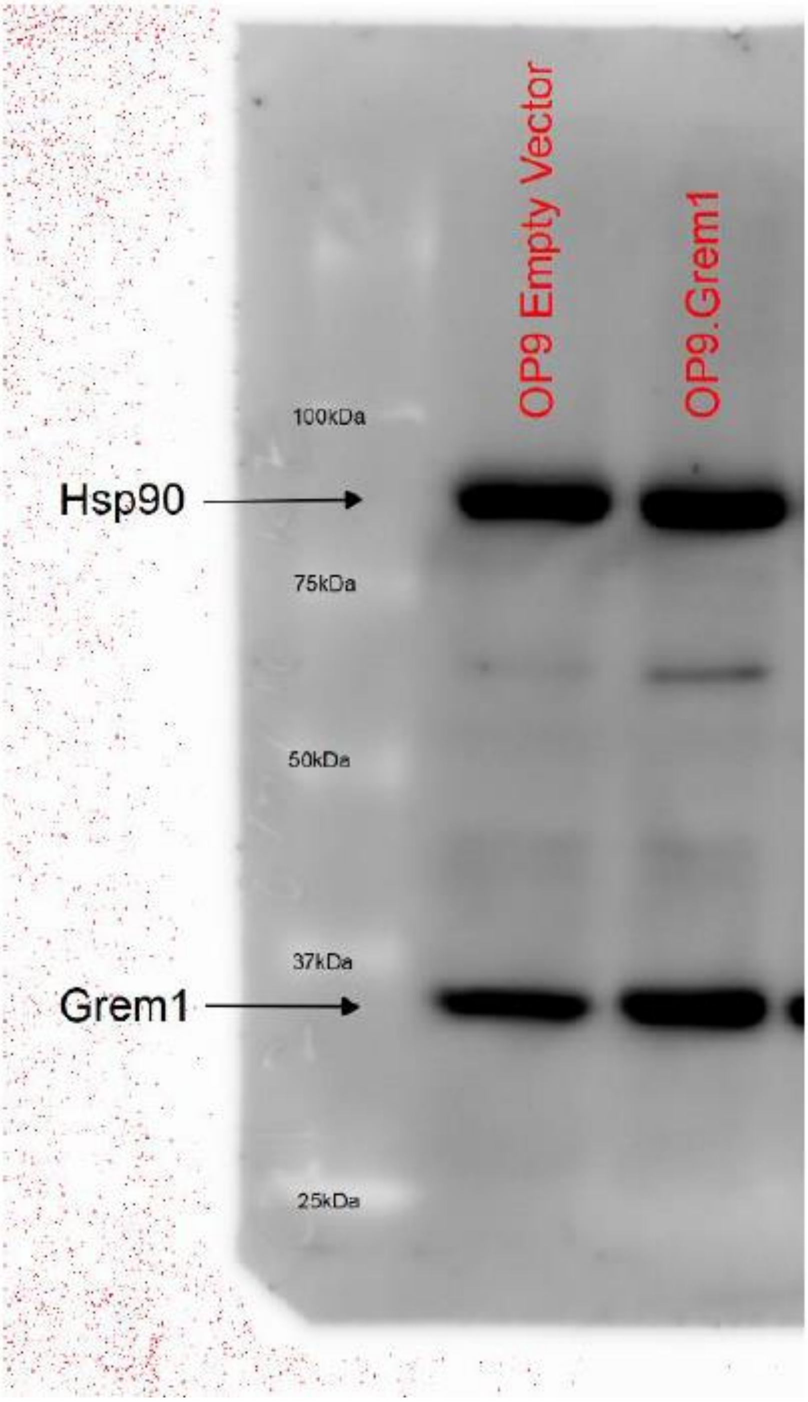

Figure S7. Grem1 full western blot.

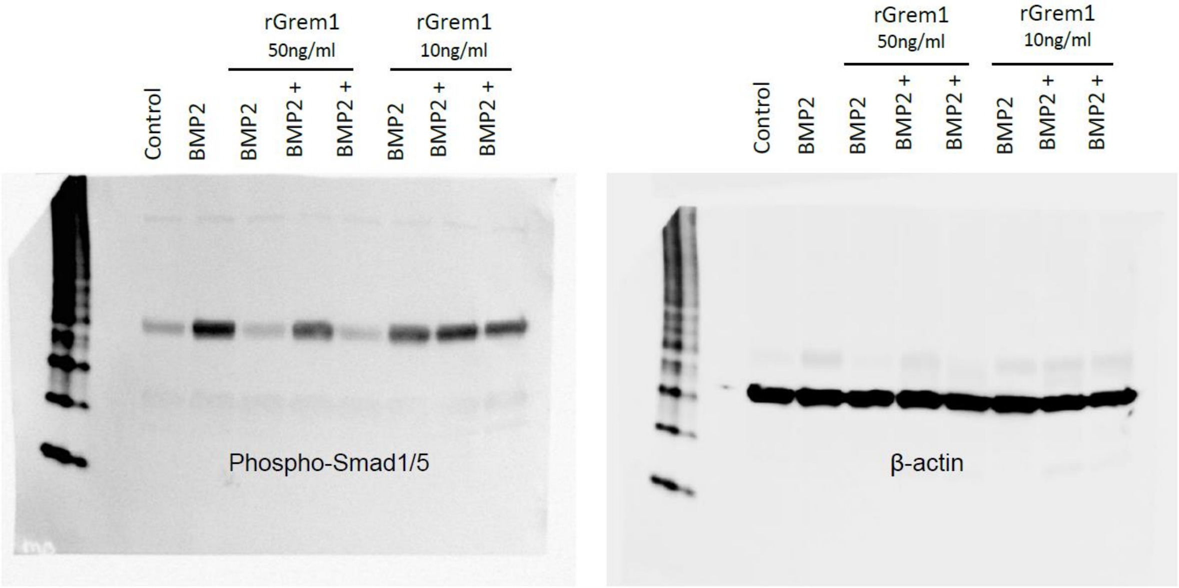

Figure S8. Phospho-Smad1/5/9 full western blot. Images from same blot taken on consecutive days to account for antibody incubation and intensity of band signals.

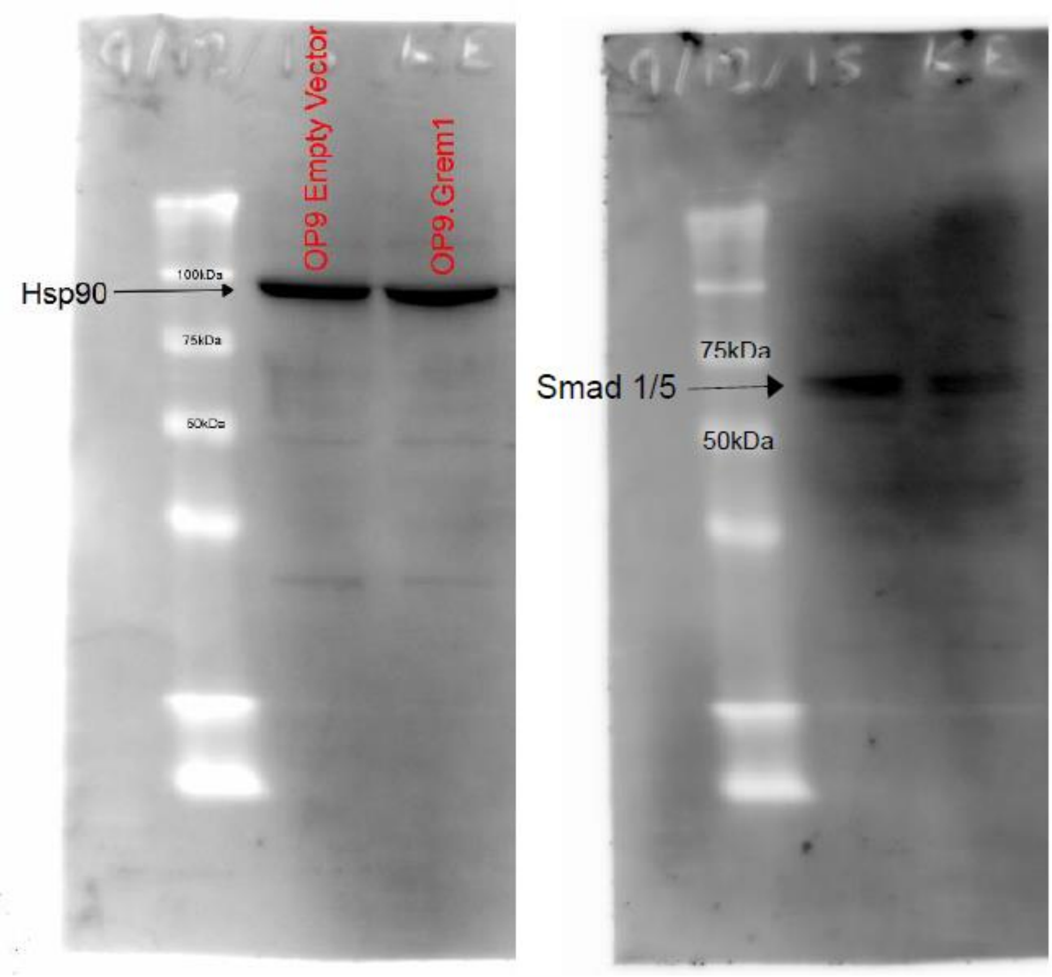

Figure S9: Phospho-Smad1/5/9 full western blot**.** Images from same blot taken on consecutive days to account for antibody incubation and intensity of band signals.

© 2020 by the authors. Licensee MDPI, Basel, Switzerland. This article is an open access article distributed under the terms and conditions of the Creative Commons Attribution (CC BY) license (http://creativecommons.org/licenses/by/4.0/).
